# Diseases of the Pancreas

**Published:** 1904-11-12

**Authors:** 


### DISEASES OF THE PANCREAS.

**Chemical Examination of the Urine.**—Changes in the urine sufficiently marked to make their detection easy by ordinary chemical methods have been described by several observers. Cammidge[1] has noted that fat necrosis, an obvious lesion in acute hæmorrhagic pancreatitis, is also probably present in less degree in all affections of the pancreas. The pancreatic ferment splits the fat into a fatty acid, or a calcium salt of a fatty acid and glycerine, the latter being absorbed and excreted in the urine. The presence of glycerine in the urine in pancreatic disease may be shown by various methods, the most practicable depending on the fact that when treated with mineral acids glycerose is formed, and may be demonstrated by a modification of the phenyl-hydrazin test used for diabetic urine. The procedure is as follows: If sugar is present it is removed by fermentation, and albumen, if necessary, is also precipitated and removed. A specimen of filtered urine is then boiled with nitric acid and then neutralised with lead carbonate. The liquid is then again filtered, and the filtrate boiled with phenyl-hydrazin hydrochloride, and sodium acetate. In pancreatic disease, and also in some other conditions characterised by active tissue changes, a precipitate of yellow crystals is formed, which may be recognised microscopically. Another specimen of urine is then treated with perchloride of mercury, and again put through the same process as the first specimen. A positive result occurs in cancer of the pancreas, and in the other conditions under which the characteristic crystals are formed apart from pancreatic disease, but a negative result in acute and chronic pancreatitis. A further differential diagnosis may be made by noting the time the crystals take to dissolve under the microscope in dilute sulphuric acid; the crystals in acute cases of pancreatitis dissolving more rapidly than those in chronic cases; while by the second method the crystals in cancer of the pancreas dissolve veiy slowly, those in old quiescent pancreatic disease more rapidly, and those in cases of extra-pancreatic disease most rapidly of all. Cammidge has altogether examined 300 specimens of urine, including controls, and believes the method reliable. He further points out that the reaction is not due to traces of lead, as has been suggested, because barium carbonate may be substituted as a neutraliser. Bushnall,[2] however, has obtained a negative result in a case proved post-mortem to be one of inter- and intra-lobular fibrosis of the pancreas. Edsall,[3] owing to the fact that in pancreatic disease the external secretion of the gland is obstructed, considers that less bacterial decomposition is likely to take place in the small intestine, as the ingested albumens are not easily broken up by micro-organisms. Hence the amount of etherial sulphates in the urine should be decreased; but the same result occurs in hyperchlorhydria and diarrhœa, or when the patient is on milk diet. Lauder Brunton,[4] moreover, points out that when a second pancreatic duct is patent this kind of test, which depends on mechanical obstruction of the common duct will not hold good. Opie[5] has described a case in which the fat-splitting ferment of the pancreas was demonstrated in the urine post-mortem by the power of the latter to decompose purified ethyl butyrate with the formation of butyric acid.

**The Pancreas and Diabetes.**—The relationship between diabetes and disease of the pancreas was noted, as pointed out by Opie[6] as early as 1788, but the absolute demonstration did not take place till v. Mering and Minkowski succeeded in completely extirpating the pancreas in dogs—an operation followed in a few hours by glycosuria. In one-third of the cases of diabetes, however, the pancreas is normal macroscopically and microscopically. The commonest lesion is interstitial pancreatitis, because the islands of Langerhans are early implicated in this condition. Ssobolew[7] has reported 17 cases of diabetes, in only two of which were the islands of Langerhans normal. Weichselbaum and Stangl[8] report 35 cases in three of which the islands were normal, but in two of these the pancreas was not thought to be affected during life. Hertzog[9] has reported five cases in which marked pancreatic disease was found, and Lepine[10] two, in both of which the islands were diseased. On the other hand Schmidt[11] out of 23 cases found the islands affected in only two, and v. Hauseman[12] out of 34 cases found normal islands in all, though in some the islands were diminished in number, and in one hyaline changes had occurred in some of the cells. He also reports a case in which fibrosis of the islands was present without diabetes. Thus out of 116 cases lesions were found in the islands in 56, and in 60 no lesions sufficient to account for the condition were observed. Allan,[13] in view of the results obtained in animals, advocates grafting of the pancreas of sheep in cases of diabetes, on the analogy of thyroid grafting for myxœdema. He admits, however, that two recorded cases have been unsuccessful, and that feeding with pancreas or pancreatic extract gives no good result.

[1] Brit. Med Jour., April 2, 1904, p. 776. [2] Ibid., June 18, 1904, p. 1463. [3] Practitioner, April 1904, p. 588. [4] Brit. Med. Jour., June 11, 1904, p. 1353. [5] Practitioner, April 1904, p. 589. [6] Med. Rec., Feb. 20, 1904, p. 313. [7] Practitioner, April 1904, p. 590. [8] Ibid. [9] Ibid., p. 591. [10] Ibid., p. 594. [11] Ibid., p. 591. [12] Ibid., p. 594. [13] Lancet, May 14, 1904, p. 1343.

*(To be continued.)*

